# Robert Henderson: Scottish doctor who was appointed Physician to the Forces (1795) and practised at Brighton, England

**DOI:** 10.1177/09677720221116550

**Published:** 2022-08-09

**Authors:** Maxwell John Cooper, Menaka Jegatheesan, Carl Fernandes, Benjamin Whiston

**Affiliations:** 1 Department of Primary Care and Public Health, 12190 Brighton and Sussex Medical School, Brighton, UK; 2 Worthing Hospital, Sussex, UK

**Keywords:** Brighton, Sussex, chalybeate, Aberdeen, Napeoleon, Trafalgar, physician

## Abstract

Robert Henderson was a Scottish physician who qualified Doctor of Medicine at Aberdeen in 1786. By 1792, Henderson was working in Brighton on the south coast of England. He was admitted Licentiate of the College of Physicians of London in 1793. At Brighton he probably worked as a parish doctor. In 1795 Henderson was appointed Physician to the Forces and probably served as a garrison doctor. In Brighton, he is noted as an advocate of chalybeate water therapy (i.e. mineral spring water containing iron salts). Henderson undertook basic experiments into the chemistry of mineral water and a few, very brief, clinical observations may be his. In Henderson's time, the chalybeate in question was part of the ‘Wick estate’ to the North West of Brighton. Today the site of the spring is located within St Ann's Well Gardens, Hove and this article briefly considers its history. Circumstances link Henderson to Sir Lucas Pepys MD (1742–1830), physician-general to the army and closely associated with both the College of Physicians and the town of Brighton. Henderson died in Brighton on the 3rd April 1808. Henderson's daughter Sophia Janet married Captain William John Thompson Hood who served at Trafalgar aged eleven.

## Introduction

This article brings together details of the life of the British army physician Robert Henderson who died in 1808. Munk's Roll of the Royal College of Physicians of London records Henderson's brief biography thus: “A native of Scotland, and a doctor of medicine of Aberdeen, of 20th May, 1786; was admitted a Licentiate of the College of Physicians 23rd December, 1793. He held the appointment of physician to the forces, and died at Brighton 3rd April, 1808”.^1^ He may be the same Robert Henderson whose ‘first year of study’ in medicine at Edinburgh University was 1783 and is recorded as an apprentice to the Royal College of Surgeons of Edinburgh in the same year.^2^ Nothing else is documented about Henderson's life prior to 1792.

## Life in Brighton (c.1792–1808)

**Image 1.**
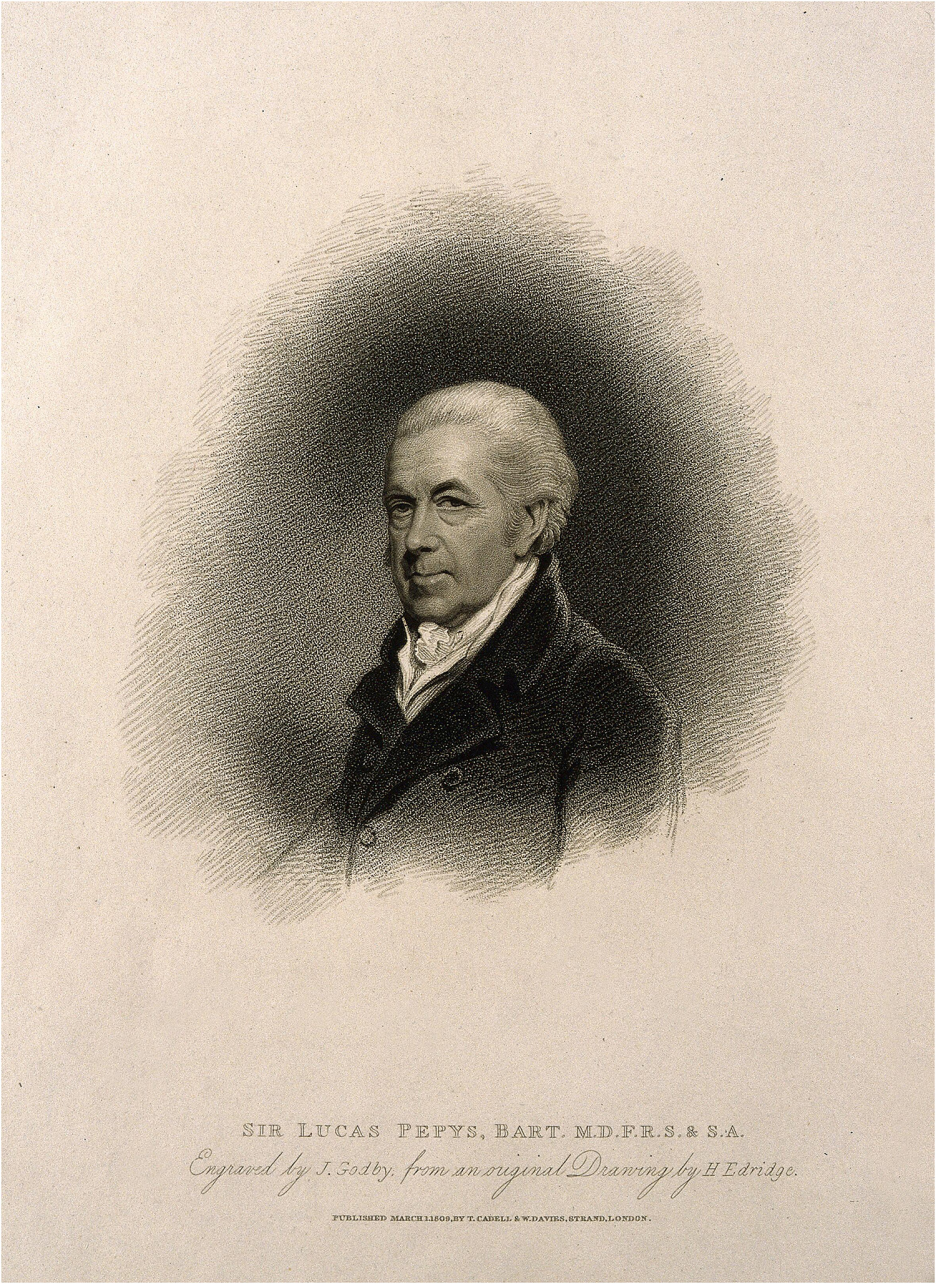
Sir Lucas Pepys (1742-1830). Pepys was appointed physician in ordinary to King George the third and physician-general to the army. His grandfather was Dr Richard Russell who practised at Lewes and Brighton. Stipple engraving by J. Godby, 1809, after H. Edridge. Courtesy of the Wellcome Collection.

By 1792 Henderson had moved to Brighton on the south coast of England. At this time, the town was popular with royalty and as a health resort for seawater therapy. The latter had been profitably promoted in Brighton by Dr Richard Russell FRS (1687–1759) in the mid-eighteenth century. Water therapy continued to be advocated by later physicians of the town, most notably Anthony Relhan (c.1715–1776) and John Awsiter (1732–1801).^3^ It is possible that a similar regime was offered there by Russell's grandson, Sir Lucas Pepys (1742–1830), who practised in Brighton during the late eighteenth and early nineteenth century (see image 1).^4^

**Image 2.**
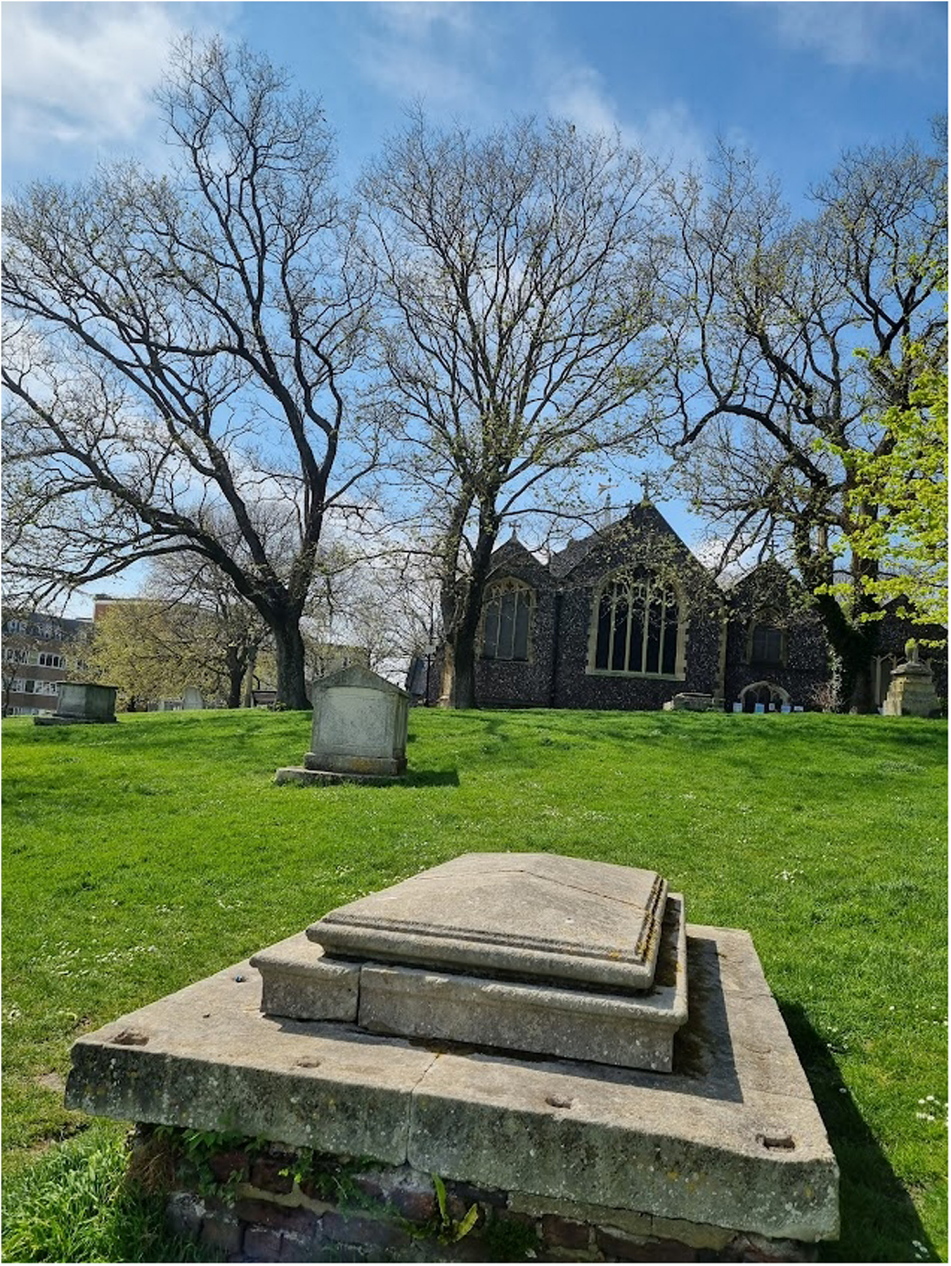
St Nicholas' Church, Brighton. St Nicholas of Myra church dates to at least the 14th century and contains a memorial to the Duke of Wellington who worshipped there in his youth. Photograph taken in May 2022 by MJF Cooper.

**Image 3.**
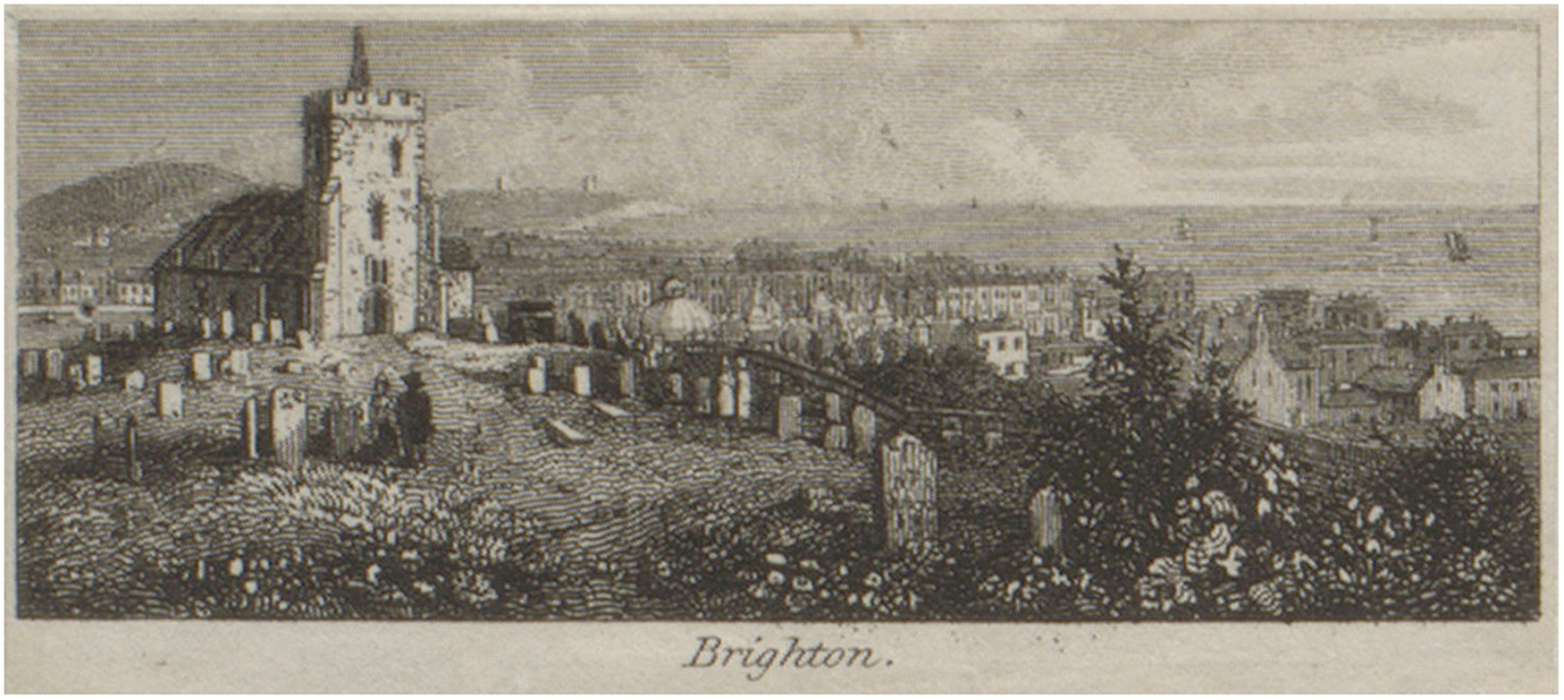
View of St Nicholas Church looking north east from the churchyard with the sea in the distance (probably c1800) Henderson's daughter was buried here. Royal Pavilion & Museums, Brighton & Hove.

The first mention of Henderson in Brighton comes from the burial records of St Nicholas’ church (see images 2 and 3). On the 18th November 1792, it is noted: “Henderson Sophia d. [daughter] of Dr Robert Henderson and his wife”.^5^

Erredge's 1862 book on the history of Brighthelmston (the original name for Brighton) also makes reference to Henderson. Therein, an account of the Hassell family mentions Henderson in December 1792:

**Image 4.**
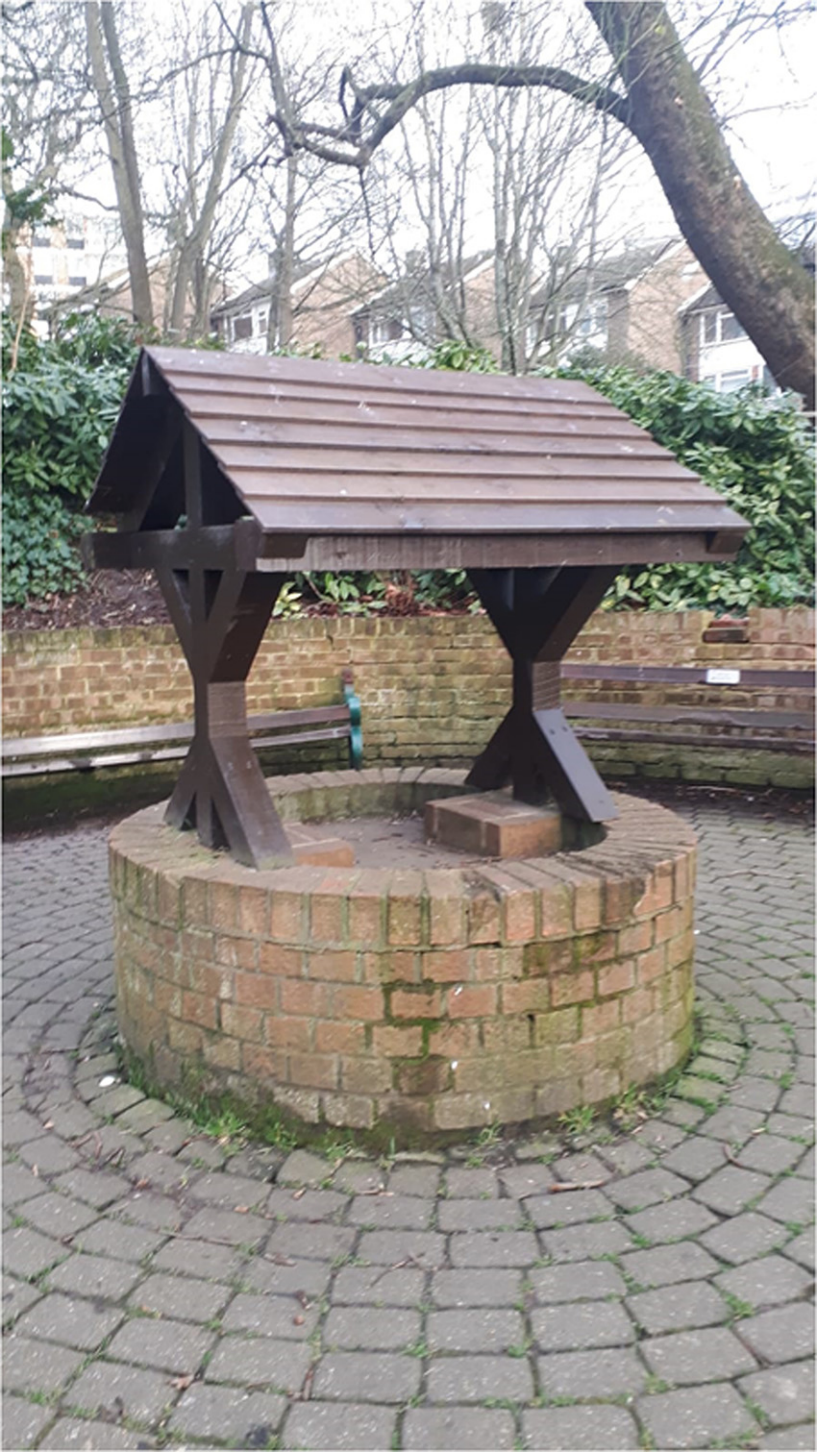
St Ann's well, Hove. This is the site of the chalybeate described by Henderson. The spring is believed to be Saxon in origin. Photograph taken in March 2022 by B Whiston.

“After but a short widowhood, she married William Hassell, of whom little is known beyond what is recorded in the parish book of Brighton; extracts from which will show that in 1792 they were in poverty, as at a meeting of the Churchwardens and Overseers, held at the Castle Tavern, on the 5th of December that year, it was:—“Ordered that Phœbe, the wife of William Hassell, be paid three guineas to get their bed and netts, which they had pledged to pay Dr Henderson for medicine.””^6^

Erredge makes two further mentions of him in 1794 which clearly refer to a single event. These suggest that he held the office of parish doctor, i.e. employed to provide medical services to its poor:

“Dr Henderson was a physician of eminence in the town; and a minute of Vestry, at the Unicorn Inn, February 10th, 1794, shews the esteem in which he was held by the inhabitants. It runs thus:—“Dr Henderson presented with a pint silver cup, for his care and attention to the parish.””^6^

^“^1794.—February 10th, Dr Henderson at a Vestry meeting, held at the Unicorn Inn, was presented with a pint silver cup, for his care and attention to the Parish.”^6^

On the 30^th^ January 1794 the baptism records of St Nicholas’ church reveal the arrival of another daughter: “HENDERSON Sophia Janet d. [daughter] of Robert M.D. and Sophia.^7^

On the 22^nd^ May 1798 the same baptism records note the birth of a son: “HENDERSON John Hardinge (b. 1.9.96) s. [son] of Robert M.D. and Sophia Elizabeth”.^7^ John's second name may indicate his mother's maiden name.

## Henderson and the chalybeate spring at ‘the Wick’

In Erredge's account of Brighton history, Henderson is noted as an advocate of chalybeate water.^6^ Chalybeate water is essentially mineral spring water containing iron salts and, from the early seventeenth century, was promoted as a health tonic in England. The primary source for the account below is unknown but Erredge may have taken it from Relhan's 1761 book entitled “A short history of Brighthelmston: with remarks on its air, and an analysis of its waters, particularly of an uncommon mineral one long discovered, though but lately used”.^8^ The description below suggests that Henderson undertook basic experiments into the chemistry of mineral water and the very brief clinical descriptions may be his. The quotation (starting with Erredge's introductory paragraph) is reproduced below in its entirety, as it appears to constitute the only words of Henderson to survive:

“Nature has been peculiarly bountiful in her goodness towards Brighton; as, independent of the salubrity of the position of the town and the superlative excellence of its sea-water, the Chalybeate spring at the Wick is possessed of great curative properties, the opinion of Drs. Russell and Relhan, being confirmed by Dr Henderson, who thus writes:—

This water, when first taken from the spring in a glass, in appearance greatly resembles a solution of emetic tartar in common water. The taste is not unpleasant, something like that upon a knife after it has been used in cutting lemons. It does not seem to contain the smallest portion of sulphur; it neither changes vegetable blues [sic], red, nor does it effervesce with alkaline salts, calcareous earths, magnesia, nor fossil alkali; neither does it change vegetable blues, green, nor does it effervesce with acids; yet it curdles soap, and renders a solution of it in various spirits milky.

It seems to contain a considerable portion of calcareous earth, mixed with vitriolic acid in the form of its selenites, and also a considerable portion of iron, as will appear from the following experiment: Sixty-four ounces of this water by measure being evaporated to dryness, there was a residuum of a brownish colour, full of spiculæ, weighing eight grains, four ounces of which, with an equal quantity of charcoal, was made into a paste with oil, and calcined. On trying the calcined matter with the magnet, two pieces nearly in the metallic form adhered to it; and when put upon paper, at the distance of half an inch, moved in every direction with the magnet. These two pieces weighed one-eighth of a grain.

The gross residuum neither effervesces with alkali nor acids, and is sufficiently soluble in water. This water becomes instantly transparent, like distilled water, on the addition of any of the mineral acids, especially the vitriolic. A solution of galls in common water, added to an equal portion of this water, becomes black like ink, in a few minutes.”^4^

Erredge then quotes the following clinical observations as a direct extension of Henderson's writing.^6^ In Relhan, however, it is unclear exactly whose words they are:

“The Chalybeate has been found serviceable in several cases of general debility, crapulas [i.e. a sense of intoxication resulting from excessive food or drink], indigestion, atony of the stomach, and fluor albus; and in all those diseases where chalybeate and tonic remedies are required, it promises, under due regulation, to be useful.”^6,8^

The passages above highlight contemporary approaches to identifying chemical composition of water: visual inspection, taste, effect on other substances (e.g. alkali and soap) and use of a magnet. It seems logical to deduce that Henderson sought to link the water's qualities with clinical observations of his patients and their symptoms. The reference to ‘due regulation’ clearly points to the individual doctor's treatment regime (which probably included instruction on diet and exercise),

This chalybeate spring later became known as St Ann's well (see image 4), which is today located within St Ann's Well Gardens in Hove. According to its website, the spring is Saxon in origin and located at the starting point of a ‘ley line’ that extends across the South Downs.^9^ Relhan describes the spring's historical origin thus:

“Some years have now elapsed since a mineral spring has been accidentally discovered to the north-west of the town of Brighthelmston, and at the distance of half a mile. The peasants who first made the discovery, though unacquainted with the virtues of its water, and even displeased with the taste of it, resolved to preserve it for the use of themselves, and the sheep which feed around it: no spring besides appearing upon the surface in all these downs, no pool of even stagnant water. The spring issues from the declining part of a little hill covered with furze; the soil around is loamy, with various strata of bole, ochre, and umbre”^8^

Elsewhere It is recorded that in the mid-eighteenth century Richard Russell “caused a valuable mineral spring at Wick, about a mile from the town, to be inclosed [sic] within a bason [sic]; and a convenient little building was afterwards erected over it”.^10^ Russell's clinical description of chalybeate waters may offer some evidence for its therapeutic value. That is because his 1755 book “The oeconomy of nature in acute and chronical diseases of the glands” advocated (with some circumlocution) the use of chalybeates in the treatment of heavy menstrual periods and cachexia (profound weight loss).^11^ Clinical improvements in both of these conditions could arise through iron supplementation, an outcome that would tally with Henderson's deduction above that chalybeates contained ‘a considerable portion of iron’.

In her article on Brighton's Georgian and Regency gardens, Sue Berry provides further information about the spring's history:

“To the west of [Brighton], in the parish of Hove, flowed the chalybeate spring at Wick. Many of the doctors who published books or articles about Brighton and most guidebooks mentioned Wick and so it received considerable publicity. In the 1750s a small building was erected at the spring and during the season a dipper was employed to offer water from it. The Scutts who owned the land improved the setting during the 1760s. In the early 1800s, Thomas Scutt redesigned the area and it was then that the colonnade on the front of the building, which features in many prints, was built. By the early 1820s the grounds had a shrubbery and a confectioner's shop, musicians played there regularly and fetes were organized”.^12^

In Henderson's day the area was part of the Wick Estate, an area of pasture and farmland which stretched from Hove village to Preston manor.^13^ The name is remembered today by the Wick Inn, Western Road, Hove. It is of interest that the Wick estate appears to have descended to Benjamin Scutt, a Brighton surgeon. In 1805 Scutt is recorded in practice with another surgeon called Hammond and working from Carleton [sic] place.^14^

Henderson is recorded as a medical practitioner at Brighton in three local guidebooks: Cobby's Brighthelmston Directory for 1799^15^ and 1800^16^ and Button's Sussex Directory of 1805.^14^ None of these supplies an address for Henderson and in both editions of Cobby he is mistakenly called ‘Richard’ rather than ‘Robert’. Reasons for this error are unclear. It may be that he was simply known as ‘Dr Henderson’ but the failure to correct his forename between editions could suggest that private practice did not constitute a significant portion of his work and income at this time. Such a deduction would align with the demands of a parish doctor and his appointment as an army physician.

## Military career

It is significant that Henderson's association with Brighton dates back to at least 1792. This is relevant because it precedes the establishment of a large garrison nearby the following year:

“In 1793, an encampment of about 10,000 troops, militia and regulars, took place at Hove, near this town, and continued, on account of some apprehensions of an invasion by the new Republic of France”.^10^

That Henderson's move to Brighton preceded his military service is confirmed by his army records. Henderson's appointment as physician to the forces was reported in the London Gazette on the 6^th^ October 1795.^17^ This is corroborated by Drew in his book of the commissioned officers in the medical services of the British Army. Drew offers the following records for Henderson's service: 5^th^ October 1795: Physician to the forces; 25 April 1798: half pay; 25th June 1802: half pay; 25th July 1803: full pay; 25th March 1805: half pay.^18^ Drew's date of death in Brighton confirms that this is indeed the same Robert Henderson. The dates of Henderson's return to full pay align with periods of threat from invasion by the French: 1794–8 and 1803–5. This would suggest that he was employed in the capacity of garrison physician.

## Evidence of association with Sir Lucas Pepys

It is of note that Henderson was already living in Brighton at the time of his licentiateship of the College of Physicians and appointment as physician to the forces. It is likely that in Brighton Henderson met Sir Lucas Pepys MD (1742–1830). As stated above, Pepys was the grandson of Richard Russell, who established Brighton's reputation as a resort for sea water therapy. Munk states that upon the death of Anthony Relhan in 1776, Pepys was the sole medical practitioner in the town.^4^ In 1794 Pepys was appointed physician-general to the army and, thus, to the Army Medical Board. In this capacity he directed the appointment of all army physicians.^4^ Munk makes the following observation about Pepys:

“Sir Lucas made his appointments, we are told by Sir James McGrigor, from the ranks of civil life, without regard to previous service in the army, and proceeding on the principle that the army physician should possess the most extensive acquirements and the most complete education, he made it a rule that all candidates for appointment should be fellows or licentiates of the College of Physicians of London, of which body he was himself, during many of the years he was at the head of the army board, the president.”^4^

These chronology of these circumstances (and Henderson's lack of military experience) clearly point to the hand of Sir Lucas Pepys in his appointment to the forces.

## Illness and death

A search of local newspapers (held on microfiche) only revealed two references to Henderson, both in relation to his death in 1808. One of these points to his suffering a ‘very long and painful illness’.^19^ This observation suggests that he may have served as parish doctor until as late as 1807. That deduction arises because in June of the same year an advertisement for the position of parish doctor was published in the Sussex Advertiser and could indicate the time when Henderson resigned from the role. It states:

“BRIGHTHELMSTON

TO THE FACULTY

NOTICE is hereby given, That the Church wardens, and Overseers of the Poor, of the parish of Brighthelmston, will meet in vestry, at the Town Hall, on Thursday the 18th instant, at eight o’clock in the evening, to receive sealed Proposals for Medicines and Attendance on the Poor of the said parish, for one year, to commence from the 24th day of June instant. It is intended that the said Tender shall include Surgery and Midwifery, and also the dispensing such prescriptions as may be ordered by any Physician, at the request of the said Parish Officers.

Further particulars may be known, by applying to Mr W. Colbron, acting Overseer; and no Tender will be received after nine o’clock in the evening"^20^

Henderson's death and burial are reported in the Sussex Advertiser in 1808. His death is not recorded in the burial records of St Nicholas’ church probably due to a gap for this year. The first quotation highlights Henderson's Christian faith. The second entry below suggests that the Henderson family vault was located inside the graveyard of St Nicholas’ church, Brighton:

“Yesterday, at his house in this town, R Henderson, Esq. M.D. after a very long and painful illness, which he supported with the firmness of a Philosopher, and the resignation of a good Christian"^19^

^“^On Saturday the remains of Dr Henderson, were interred in his family vault, in the cemetery of this parish”.^21^

A search of St Nicholas’ graveyard and its two extensions revealed no tombstone bearing the name Henderson. Some altar tombs and perimeter tombstones do remain in the graveyard today (see image 3). However, the inscriptions upon many gravestones have worn away over time and clearances at the sites took place in the mid-twentieth century. No record of the Henderson vault could be found in a search of inscription and monument records held at the county archives.

## Family members and association with Captain William John Thompson Hood

No entry for Henderson's wife, Sophia, was identified in Brighton burial records. It is possible that after Henderson's death, she moved back to her original family home. Given a contemporary association of her son's middle name Harding(e) with the West of England,^22^ this could have been to the county of Somerset. A record of such circumstances does exist at St Swithin's church, Walcot, Bath in that county. Its burial register states: “Sophia Elizabeth Henderson, aged 73, of 4 Bennett Street, was buried on 15 Aug 1838”.^23^ Sophia's monument in the crypt of St Saviour's church, Bath, offers additional information about her husband that might support this deduction:

“IN THE CRYPT BENEATH LIE THE REMAINS OF SOPHIA ELIZABETH, RELICT OF THE LATE ROBERT HENDERSON THIS TRIBUTE OF AFFECTIONS TO HER MEMORY IF ERECTED BY HE AFFECTIONATE DAUGHTERS SHE DEPARTED THIS LIFE AUGUST 7TH 1838 AGED 73”^18^

In 1830 Henderson's daughter Sophia Janet married Captain William John Thompson Hood, Royal Navy, who had served at the battle of Trafalgar aboard HMS Achille aged 11.^24^ His biography ends thus:

“Capt. Hood, on 26 May, 1824, received from the Society for the Encouragement of Arts, &c., its gold Vulcan medal for his improved screen-glasses for quadrants and sextants for naval use. He has also been voted the silver medal of the same body for his invention of an ice-saw, for facilitating the progress or escape of ships navigating the high Polar latitudes, when surrounded by field-ice. In 1828 he obtained a similar honorary reward for his method of constructing a floating bridge, from the materials to be found on board all ships of war and vessels generally. In 1830 he again received the Society's medal for his invention of an improved rocket-shaft. Capt. Hood has also invented a rotatory lifting and forcing pump. He married, 16 Dec. 1830, Sophia Janet, second daughter of the late Robt. Henderson, Esq., Physician and Inspector [sic] of the Forces. AGENTS – Messrs. Stilwell.”^24^

## Conclusion

Little has been written about the life of Henderson beyond Munk's and Drew's brief biographical notes. Nothing is known about his early life in Scotland or motivations for moving to England. Only six years after qualifying MD at Aberdeen, Henderson had settled in Brighton. His original purpose in residing there was probably to establish himself in private practice. Such a decision is consistent with Brighton's popularity, expanding population and association with royalty. His work appears to have commenced as a parish doctor. Brighton guidebooks confirm that he also offered private services, presumably as an extension of his other roles. Given evidence of Henderson's close association with the church and the existence of a newspaper advertisement for a parish doctor, published in June 1807, it is possible that he held this office until that time.

Henderson's move to Brighton precedes Napoleon's 1793 declaration of war on Britain, an event that transformed Brighton into an important garrison town. It is possible that Pepys may have had a hand in Henderson's move to Brighton and his decision to seek the licentiateship of the college of physicians. Pepys’ role in Henderson's appointment to the forces is cemented by circumstances. There is no evidence that Henderson saw action with the army and his role appears to have been that of garrison physician. The Henderson family is associated further with the struggle against Napoleon through his son in law, Captain Hood, who had fought at Trafalgar as a boy.

Henderson undertook basic experiments into the chemistry of mineral water and some brief mention of clinical experience of it may be his. Robert Henderson's name is forgotten today in Brighton. This article aims to remedy that perspective by documenting his place as a member of a group of notable physicians who practised water therapy in the town.
